# The Radiographic Appearances of Bilateral Ureteroceles and Their Management in an Adult Patient Presenting With Recurrent Urinary Tract Infections

**DOI:** 10.7759/cureus.53487

**Published:** 2024-02-03

**Authors:** Adrian Chan, Nicolette Cassim, Jason Diljohn, Fidel S Rampersad, Devendra H Ojar

**Affiliations:** 1 Department of Radiology, The University of the West Indies, St. Augustine, TTO

**Keywords:** multi-modality imaging, ct urogram, fluroscopy, urology imaging, ureterocele

## Abstract

A ureterocele is a congenital abnormality in which there is abnormal dilatation of the distalmost portion of the ureter, as it enters the urinary bladder. Patients present with frequent urinary tract infections, urinary retention, cyclical abdominal pains, failure to thrive, and hematuria. Ureteroceles are often diagnosed on antenatal ultrasound and sometimes postnatally on ultrasounds done in the setting of a urinary tract infection.

This case describes a 51-year-old female who presented with recurrent urinary tract infections. Subsequent imaging with ultrasound, intravenous urogram, and computed tomography demonstrated features typical for bilateral ureteroceles.

## Introduction

A ureterocele is a congenital abnormality where the ureter is dilated as it enters the urinary bladder, resulting in a sac-like pouch [1,2]. Patients may present with frequent urinary tract infections, urinary retention, cyclical abdominal pains, failure to thrive, and hematuria [1,2].

Its diagnosis is based on radiological findings, with most cases being diagnosed on antenatal ultrasound. Ultrasonography is useful as a first-line investigation, demonstrating a cystic structure arising from the posterior wall of the urinary bladder [3]. Herein we report a case of bilateral ureteroceles in a patient with recurrent urinary tract infections.

## Case presentation

**Figure 1:**
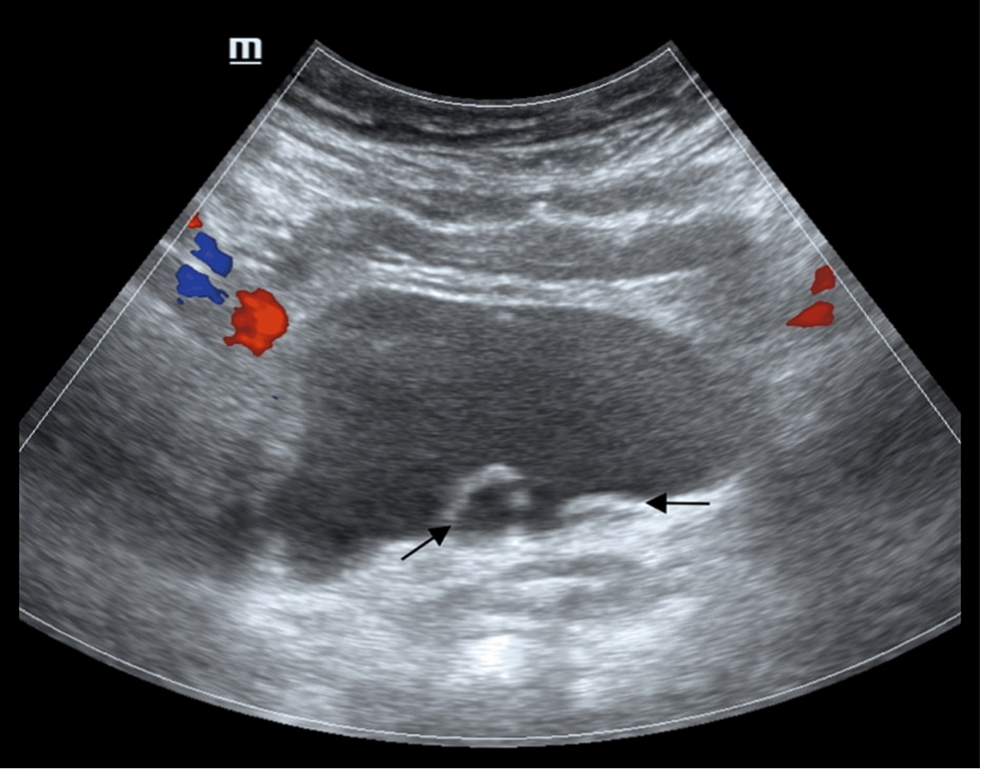
Greyscale transverse ultrasound image with color Doppler showing dilated ureters bilaterally projecting into the bladder lumen (arrows). Ureterocele is more pronounced on the right and mild on the left.

A 51-year-old female presented to the Adult Emergency Department with dysuria, urinary urgency, and frequency. Her past medical history noted recurrent urinary tract infections since childhood. Complete blood count demonstrated a mildly elevated white blood cell count of 12.3 x 10^3^/uL. The renal function test was normal. Urinalysis demonstrated leucocyte esterase 3+, nitrite 3+, protein +, and trace blood. Her urine culture was positive for *Escherichia coli*. The patient was referred to the Urology Department. Initial imaging with ultrasound showed dilated distal ureters bilaterally projecting into the urinary bladder lumen (Figure 1).

**Figure 2:**
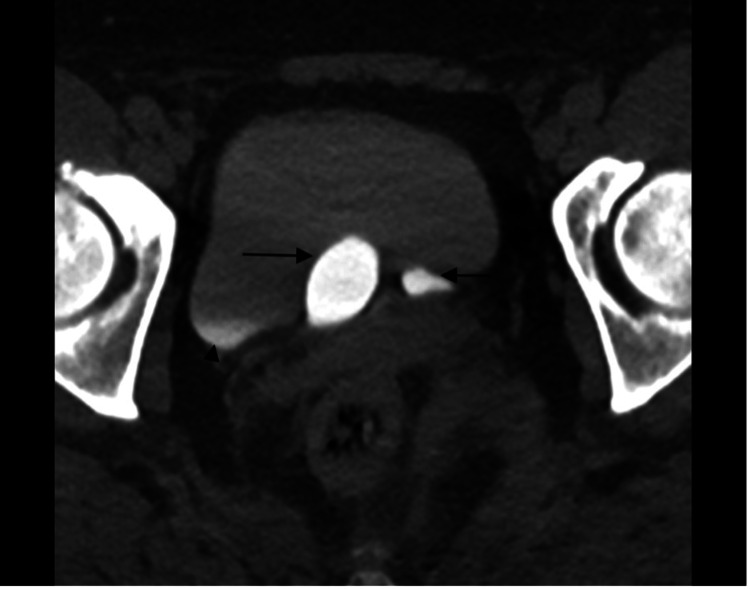
Axial IV contrast-enhanced CT image through the pelvis in the delayed urographic phase shows two well-circumscribed structures at the bladder trigone (arrows) which are opacified with contrast. A small amount of excreted contrast is also seen in the dependent portion of the urinary bladder lumen (arrowhead).

**Figure 3:**
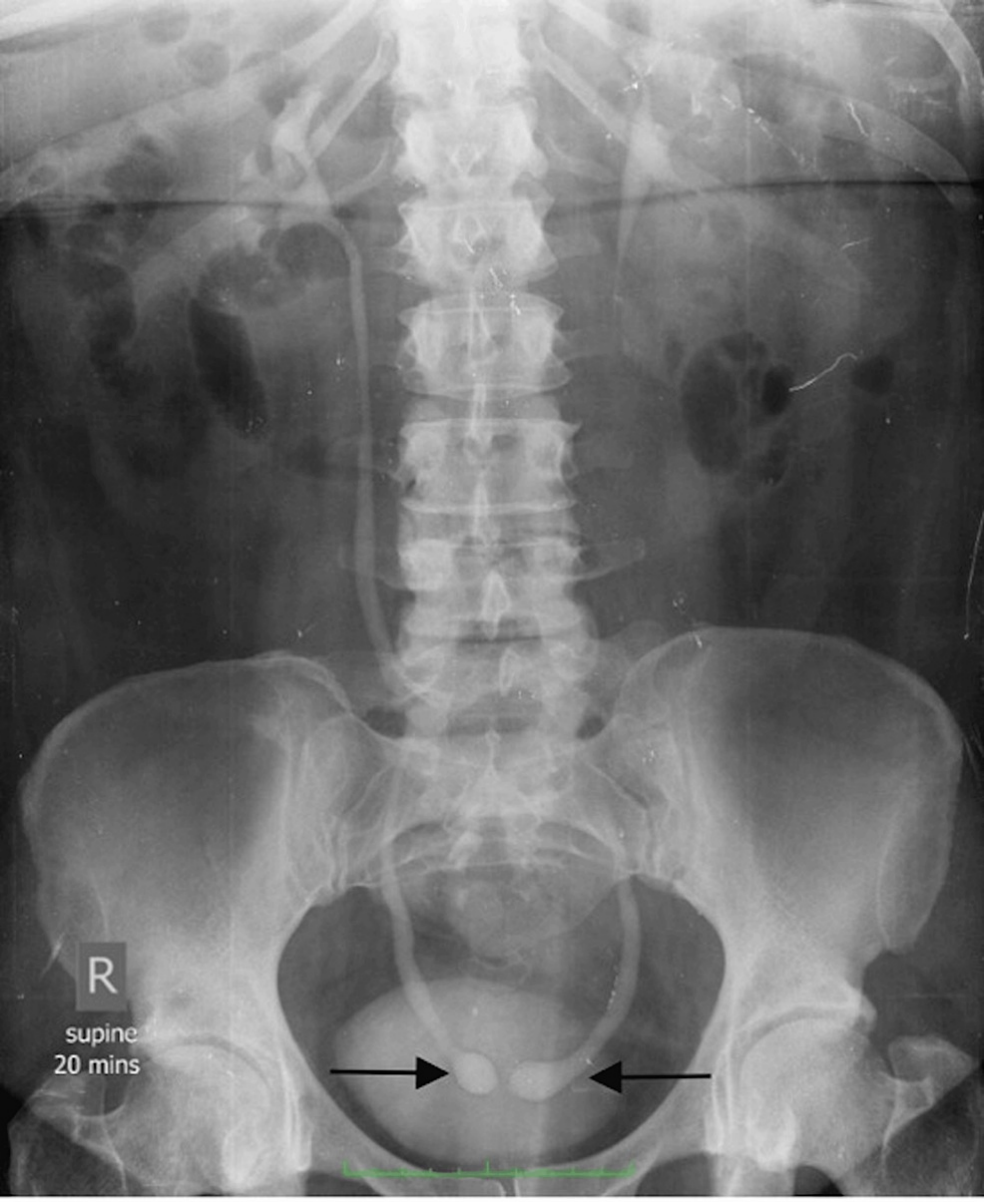
Supine AP film (20-minute film) from an intravenous urogram (IVU) demonstrating bilateral well-circumscribed dilations of the distal ureters with thin lucent rims surrounding the distal ureters (arrows). “Columnization” of contrast in the bilateral ureters is noted, proximal to the ureteroceles. There is no hydronephrosis. AP: Anteroposterior

Axial IV contrast-enhanced computed tomography (CT) scan of the abdomen and pelvis was then performed, which further demonstrated two well-circumscribed cystic structures at the bladder trigone (arrows) (Figures 2, 3).

**Figure 4:**
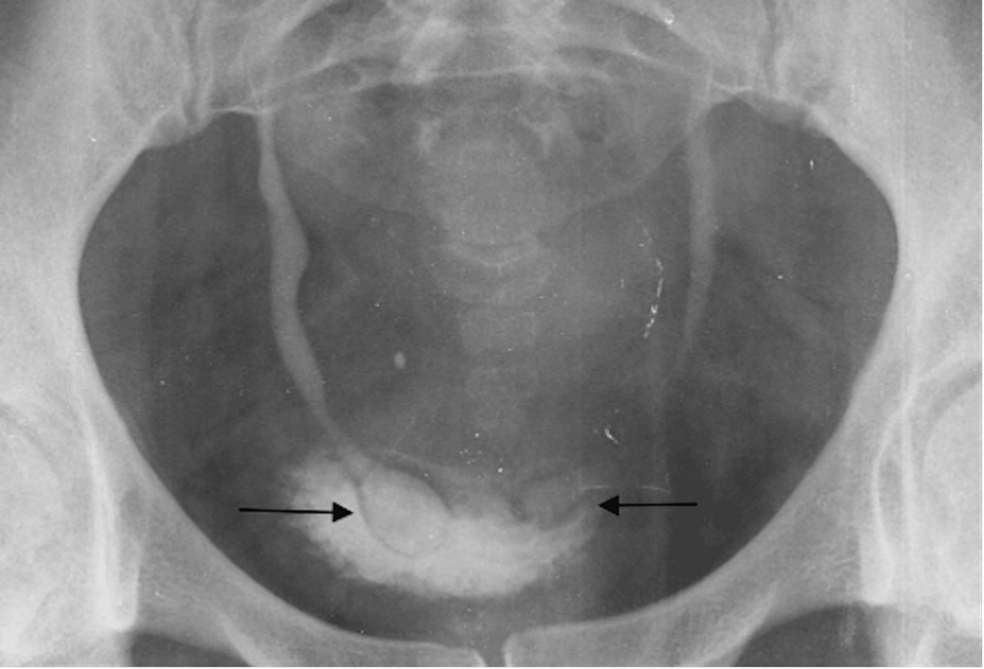
Supine AP film (post-micturition film) from an intravenous urogram shows ovoid-shaped dilation of the distal ureters at the vesicoureteric junctions, projecting into the urinary bladder lumen, with thin radiolucent rims, and post-micturition contrast holdup in the distal ureters. AP: Anteroposterior

The patient underwent further evaluation with an intravenous urethrogram (IVU) (Figure 4), in which a supine anteroposterior film (20-minute post-IV contrast injection film) showed bilateral "cobra head" appearances of the distal ureters consistent with bilateral ureteroceles.

**Figure 5:**
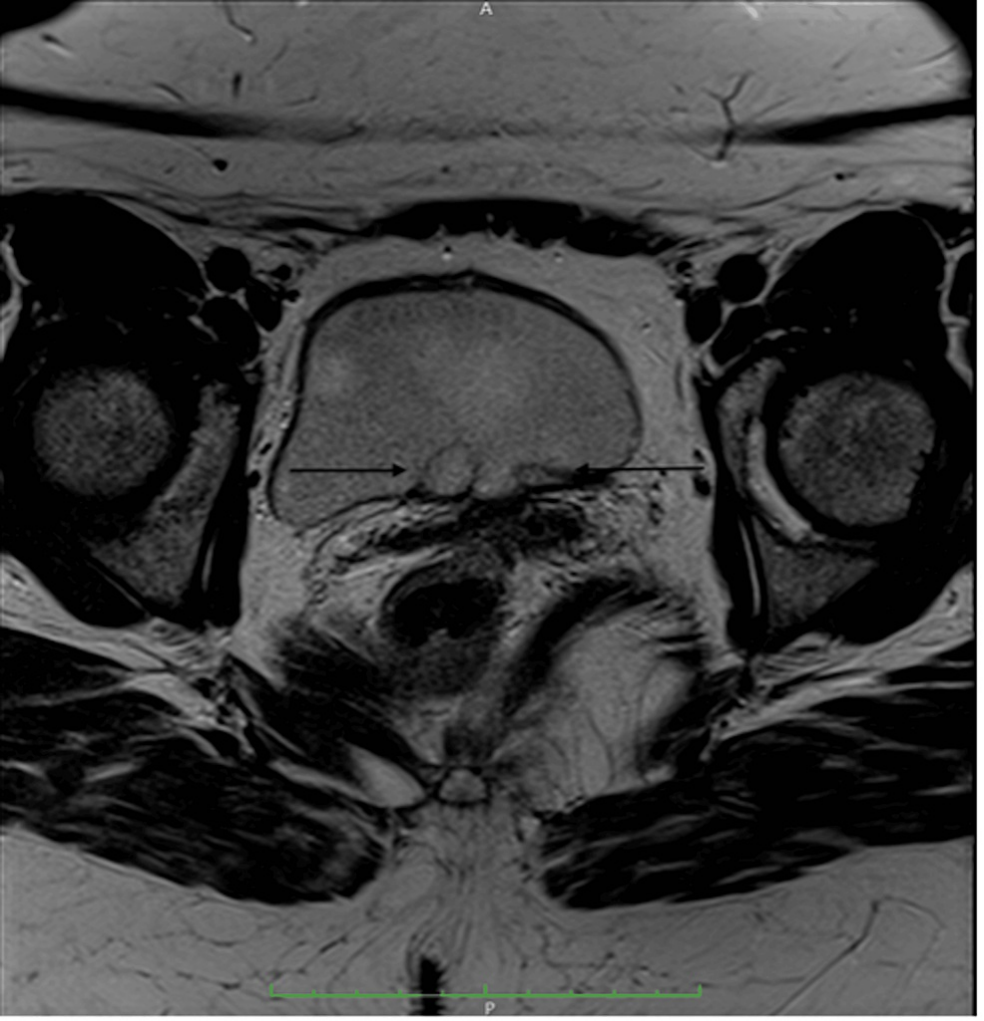
Axial T2 weighted MRI image through the pelvis showing two well-circumscribed structures at the bladder trigone (arrow) which demonstrate a thin rim of low T2 signal with central high T2 signal.

Incidentally, on performing an MRI of the pelvis and lumbar spine for an unrelated indication, these abnormalities of the distal ureters were visible on axial T2 weighted images (Figure 5), showing a similar oval dilatation of the distal ureters at the vesicoureteric junctions (VUJs) projecting into the bladder lumen.

Subsequently, a cystoscopy was performed and the presence of bilateral ureteroceles was confirmed. In our case, treatment via endoscopic puncture at the time of cystoscopy was advised however, the patient refused intervention at this time. She was therefore managed conservatively, with a course of antibiotics for her urinary tract infection. She has yearly scheduled follow-up visits, as well as open appointments at the urology outpatient clinic, where complications are monitored and treated. In our case, these complications are recurrent urinary tract infections. The patient is also counseled on the need for definitive treatment via endoscopic puncture at these visits.

## Discussion

A ureterocele is a congenital abnormality where the distalmost portion of the ureter is dilated as it enters the urinary bladder, resulting in a sac-like pouch [1,2]. The etiology of ureteroceles remains unknown. However, some studies have revealed that there may be a genetic predisposition for ureteroceles. It is postulated to be caused by failure of regression of the Chawalla membrane during embryological development [4]. The prevalence ranges from 1 in 500 to 1 in 4000 patients, with the majority (up to four-fifths) of patients being female [1,2].

Patients may present with frequent urinary tract infections, urinary retention, cyclical abdominal pains, failure to thrive, and haematuria [1,2]. There are two types of ureteroceles: simple (intravesical) and ectopic (extravesical). Simple ureteroceles occur in normally positioned ureteral orifices, with stenosis at the distal end resulting in ballooning of the segment proximal to the stenosis. These are less common and occur in ~25% of cases. Ectopic ureteroceles occur in the presence of an ectopic ureter, where there is a protrusion of the distal ureteric segment into the lumen or neck of the urinary bladder or the urethra. Ectopic ureteroceles are frequently seen in duplex kidneys with complete ureteral duplication, where the upper pole moiety ureter inserts ectopically into the urinary bladder, inferior and medial to the lower pole moiety ureter, which has an orthotopic insertion (Weigert-Meyer law) [3].

Ureteroceles can also be classified based on their size and location of the ureteric orifice. The Stephens classification describes four categories of ureterocele: stenotic, with a narrow orifice within the bladder; sphincteric, with a wide orifice within the internal sphincter; sphinctero-stenotic, with a narrow orifice within the internal sphincter; and caeco-ureterocele, in which there is a blind-ending ureterocele extending down the urethra [5].

The diagnosis is based on radiological findings, with most cases being diagnosed on antenatal ultrasound. Postnatally, ultrasonography is useful as a first-line investigation, demonstrating a cystic structure arising from the posterior wall of the urinary bladder [3].

Other imaging modalities include intravenous urography which reveals the characteristic “cobra-head sign” described as a halo produced by the wall of the ureter surrounded by contrast opacified urine. Poor function of the affected side can be manifested on IVU with delayed excretion or absence of excretion [6,7]. Contrast-enhanced CT with the use of the delayed phases and MRI using heavily T2-weighted sequences can also be used to demonstrate the size and morphology of a ureterocele and characterize its relation to the VUJ [1,7].

The management of ureteroceles varies depending on the patient’s age, presenting complaints, presence of vesicoureteric reflux, the functional capacity of each renal segment (in cases of duplex collecting systems), and presence of complications such as urinary tract infections. The primary goal of treatment is the relief of obstruction and preservation of renal function [1]. Management may be conservative or surgical. In the pediatric population, asymptomatic patients antenatally diagnosed may be managed conservatively. However, there is insufficient data regarding this approach, and it is associated with longer follow-up. It therefore is not considered to be a judicious option [8]. In adults, incidentally detected ureteroceles usually do not require treatment unless they are complicated by calculi [8]. For patients in whom the surgical approach is considered, antibiotic prophylaxis is usually given until surgery can be done. Ureteric stenting may also provide short-term relief [1]. In our case, treatment via endoscopic puncture was advised however, the patient refused intervention at this time. She was therefore managed conservatively, with a course of antibiotics for her urinary tract infection, and is now followed up in the urology outpatient clinic.

Endoscopic incision of ureterocele proves to be the definitive treatment in most of the children [8]. Primary endoscopic ureterocele puncture can also be performed in children with clinically significant ureterocoeles. Deroofing with ureteric stenting may be performed via endoscopy for an obstructing ureterocele, and has been shown to be the best initial approach for adequate decompression, with reduced rates of secondary surgery [1]. Endoscopic transurethral puncture and transurethral incision can provide a curative rate of up to 90% [3]. Non-endoscopic surgical options, including heminephrectomy and partial ureterectomy, ureteroureterostomy, or ureteropyelostomy may be used for complex cases [1].

## Conclusions

A ureterocele is a congenital abnormality where the ureter is dilated as it enters the urinary bladder, resulting in a sac-like pouch. Clinically, patients present with frequent urinary tract infections, urinary retention, cyclical abdominal pains, failure to thrive, and hematuria. The diagnosis is based on radiological findings, with most cases being diagnosed on ultrasound. Management may be conservative or surgical, with the primary goals of treatment being relief of obstruction and preservation of renal function. In adults, incidentally detected ureteroceles usually do not require treatment, unless they are complicated.
